# Feasibility and implementation of community-based malaria case management with integrated vector control in the Democratic Republic of Congo

**DOI:** 10.1186/s12936-016-1475-3

**Published:** 2016-08-15

**Authors:** Edouard Kawawa Swana, Ghislain Yav Makan, Clarence Kaut Mukeng, Henriette Ilunga Mupumba, Gabriel Mutabusha Kalaba, Oscar Numbi Luboya, Michael J. Bangs

**Affiliations:** 1 Freeport/International SOS Public Health Program, Tenke Fungurume Mining Project, Lualaba, Democratic Republic of the Congo; 2 Division Provinciale de la Santé Katanga, Lubumbashi, Democratic Republic of the Congo; 3 Faculty of Social Sciences, University of Lubumbashi, Lubumbashi, Democratic Republic of the Congo; 4 Faculty of Medicine and School of Public Health, University of Lubumbashi, Lubumbashi, Democratic Republic of the Congo; 5 Freeport/International SOS Public Health & Malaria Control, Papua, Kuala Kencana 99920 Indonesia

**Keywords:** Malaria case management, Community health workers, Integrated vector control programme, Democratic Republic of the Congo

## Abstract

**Background:**

Malaria prevalence in the Mulumbu Health Area in Lualaba Province, Democratic Republic of the Congo has remained high (>70 %) despite repeated vector control (indoor residual spray) and mass insecticide-treated bed net coverage. Therefore, a pilot study was implemented to attack the parasite directly and demonstrate the feasibility and acceptability of community case management of malaria (CCMm) using trained community health workers (CHWs).

**Methods:**

A 13 month prospective evaluation of CCMm was undertaken in 14 rural villages. Focus group discussions and structured interviews were conducted in pre- and post-intervention periods to assess community acceptability of CCMm. Weekly data collected by CHWs assessed program impact over time, matched with malaria school-based prevalence surveys (MSPS) in the Mulumbu Health Area (CCMm study arm) compared to a comparison (non-CCMm) arm in the Mpala Health Area approximately 25 km apart.

**Results:**

Overall population perception of the CCMm was highly positive. 6619 community contacts were managed by CHWs from which 1433 (21.6 %) were malaria positive by rapid detection tests during the 10 month intervention. Among the malaria infected, 94.7 % (1358) were recorded as ‘uncomplicated’ infections with 99.7 % provided full course of treatment. CHWs referred 278 (4.2 %) patients deemed ‘complicated’ to a designated primary health center for advanced care. While pre-intervention MSPS data revealed significantly higher (*p* = 0.0135) malaria in the CCMm area compared to the non-CCMm area, at post-intervention there was no statistical difference (*p* = 0.562) between the two areas. Notably, for the first time, no malaria-related deaths were recorded in the 14 CCMm intervention villages during observation.

**Conclusion:**

Community case management of malaria was shown to be an effective and promising strategy for prompt and effective management of malaria. It was well accepted by the community and showed evidence of a reduction in malaria morbidity and mortality. Further refinement of CCMm implementation, cost implications and sustainability is advised before expanding the programme.

## Background

Malaria remains a major public health burden in many endemic regions of the world and responsible for high rates of morbidity and mortality in sub-Saharan Africa. Although the number of malaria cases globally decreased from an estimated 262 million infections in 2000 (range 205–316 million), to 214 million in 2015 (range 149–303 million), a decline of 18 %, most cases (88 %) and deaths (90 %) in 2015 were confined to the WHO African Region. Together, the Democratic Republic of the Congo (DRC) and Nigeria accounted for more than 35 % of the global malaria deaths [1]. The use of long-lasting insecticidal nets (LLINs) and repeated application of residual insecticides on indoor wall surfaces remain the primary methods for vector control to reduce human-vector contact, while accurate diagnostic testing and prompt use of effective malaria treatment using artemisinin-based combination therapy (ACT) are the current hallmarks of comprehensive, integrated malaria control interventions in malaria endemic areas of Africa [2].

In 2007, a private company, Tenke Fungurume Mining (TFM), an affiliate of Freeport McMoRan Inc., began a major investment and operational footprint in southeastern Katanga Province (now Lualaba Province), DRC. The company also made an early commitment to reduce malaria morbidity and mortality by implementation of an integrated malaria control programme in both the resident workforce population and local surrounding communities consisting of Tenke and Fungurume townships and majority of smaller rural villages within the administrative purview of the Fungurume Health Zone. The company also entered into a close partnership with the government of the DRC aligning its malaria control programme with the National Malaria Control Programme (NMCP) strategic approach. By 2015, the programme was protecting approximately 200,000 people.

**Fig. 1:**
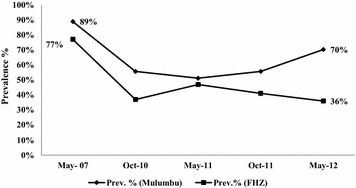
School-based malaria prevalence in Mulumbu Health Area compared to overall Fungurume Health Zone (FHZ) under vector control in the pre-intervention study phase, May 2007–2012

The TFM vector control component includes twice yearly indoor residual spraying (IRS) of insecticides on interior wall surfaces and eave areas, initial and periodic mass redistribution of LLINs, and selective larva monitoring and control using environmental management practices and/or larvicidal agents as appropriate. Following the expanded implementation and control coverage of the community programme in 2009, school-based malaria (as a monitoring tool of this programme) decreased from a pre-control prevalence of 77 % in 2007 to 39 % in May 2012, a mean reduction of around 50 % across the concession under control coverage. Moreover, the May 2012 area-wide school surveys indicated that the malaria prevalence remained particularly persistent problem in the rural Mulumbu Health Area (HA), compared to other administrative HAs under IRS coverage (unpublished data): 70.3 % in Mulumbu HA versus mean of 36 % in all other areas (Fig. 1). The high malaria prevalence was present despite excellent IRS coverage (>90 % of recorded structures) of homes and wide-spread distribution of LLINs in the Mulumbu HA. Thus, these control methods alone were deemed insufficient to curb the majority of transmission risk. Several explanations, among them the greater movement and extended stay of community members to areas outside normal residence, for example to tend subsistence gardens, are believed the principal reasons for the poor impact of intervention measures. These findings indicated that additional methods would be required to reduce malaria prevalence in Mulumbu HA in line with health areas experiencing less infection.

Access to prompt diagnosis and effective treatment is an essential component in integrated malaria control strategies. Various studies have demonstrated the feasibility and cost-effectiveness of community case management of malaria (CCMm), also known as home-based management of malaria [3–11]. WHO currently recommends CCMm using an ACT based on results from use of a high quality rapid diagnostic test (RDTs) and/or standard expert microscopy. This would greatly improve access to prompt and effective malaria case management among populations with limited access to formal health care facilities [10–12]. Therefore, a pilot study was designed to demonstrate the feasibility and utility of CCMm as part of an integrated malaria and vector control programme in remote villages using trained and supervised volunteer Community Health Workers (CHWs). The study aims were as much about the ‘process’ of programme implementation as it was showing actual outcome (as measured from pre- to post-intervention prevalence rates). Various qualitative studies (focus group discussions and interviews) were designed to assess the perceptions and acceptance of the CCMm by key community members, information that would help to refine and improve future implementation and impact.

## Methods

### Study sites

**Fig. 2:**
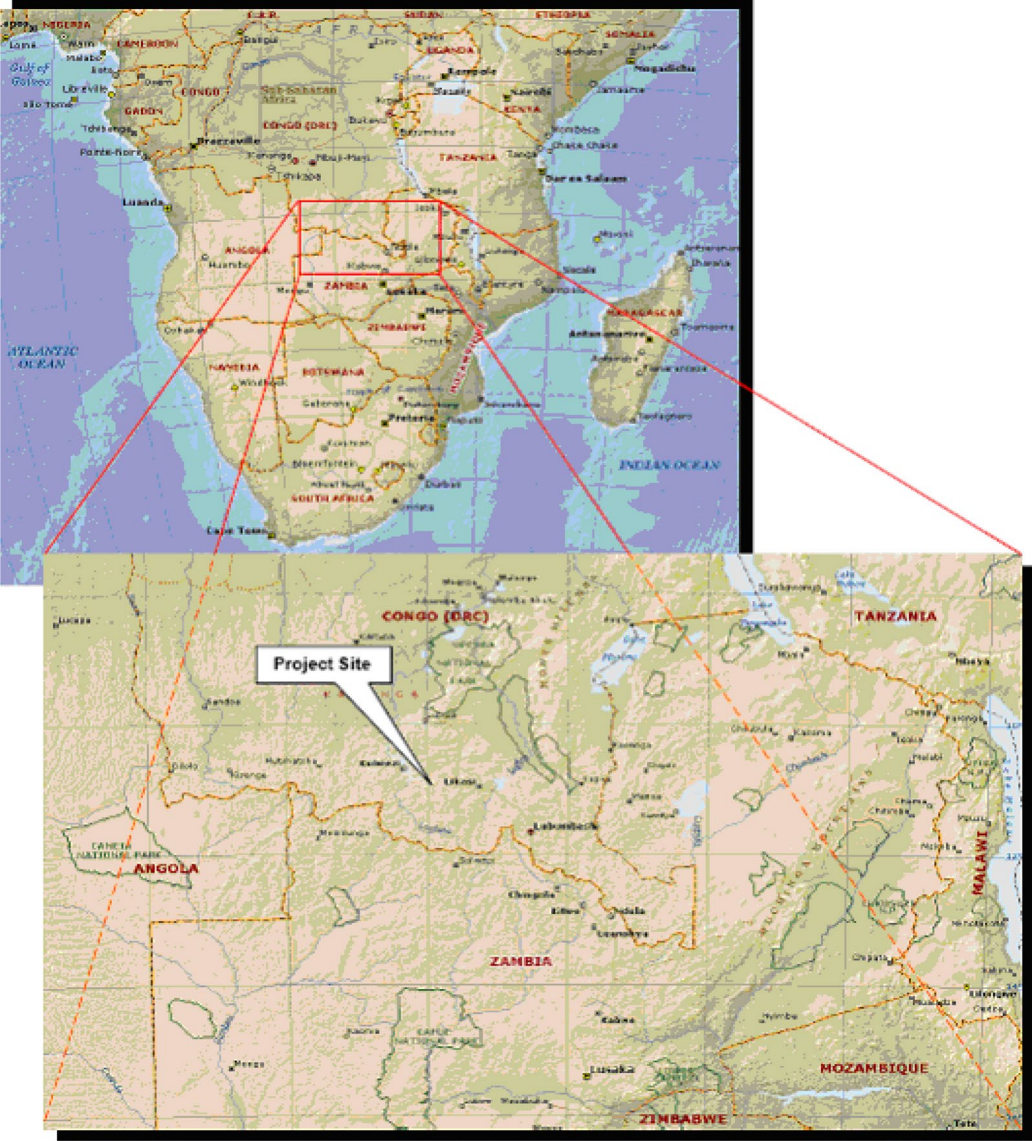
Tenke Fungurume Mining concession area in southern DRC, ~180 km North-West of Lubumbashi, capital of Haut-Katanga Province and 70 km east from Kolwezi, administrative center of Lualaba Province

The Mulumbu HA, located within the Fungurume Health Zone (FHZ), Lualaba Province (formerly Katanga Province), was selected for prospective evaluation of the implementation of the CCMm in conjunction with existing integrated vector control measures. The FHZ is one of the 14 health zones in the province. It is located approximately 185 km northwest of Lubumbashi, the administrative capital of the Haut Katanga Province (Fig. 2). With an estimated population of 273,000 inhabitants (2015), approximately 200,000 residing within TFM concession (i.e., malaria control coverage) boundaries, the FHZ is divided into 18 ‘Health Areas’.

The study was divided into an intervention and a comparison (non-CCMm intervention) arm. The approximate distance between villages of both study arms is 25 km. The intervention arm included 14 discreet rural villages in Mulumbu HA, an area with perennial malaria transmission. The Mulumbu HA has two functional health clinics supported by the mine company, with one designated the Primary Health Centre (PHC) for the area. DRC-qualified, trained nurses, two primary schools with respective headmasters and teachers, and a cadre of CHWs were available to participate. CHWs were community-based volunteers, most often selected by village elders and peers in each village. CHWs do not receive a direct salary from the local government or Ministry of Health. However, during specific activities such as mass vaccination campaigns, they receive a modest per diem to cover expenses. The intervention area had an approximate population of 3343 inhabitants and 688 households at the beginning of the study. Together with the CCMm, the villages also received periodic IRS and larval source management (LSM) activities during the study. LLINs were already in place in each village and no additional nets were provided during the study period.

The Mpala HA was selected as the non-CCMm comparison area. This rural area was deemed similar to Mulumbu by also having a history of documented high perennial malaria transmission regards background transmission risk based on consecutive biannual school-aged children malaria prevalence rates in each area. Based on the May 2012 school survey, Mpala had a combined malaria prevalence rate of 52 % [range 43.2–60.6]. Mpala consisted of six primary villages including one village population relocated from the active operational mine area, one health clinic integrated into the FHZ system as the Primary Health Centre in the area. During the study, Mpala continued to receive the standard malaria control interventions of IRS and LSM practices. No additional LLINs were provided to this population during the observation period.

### Pilot study design

**Fig. 3:**
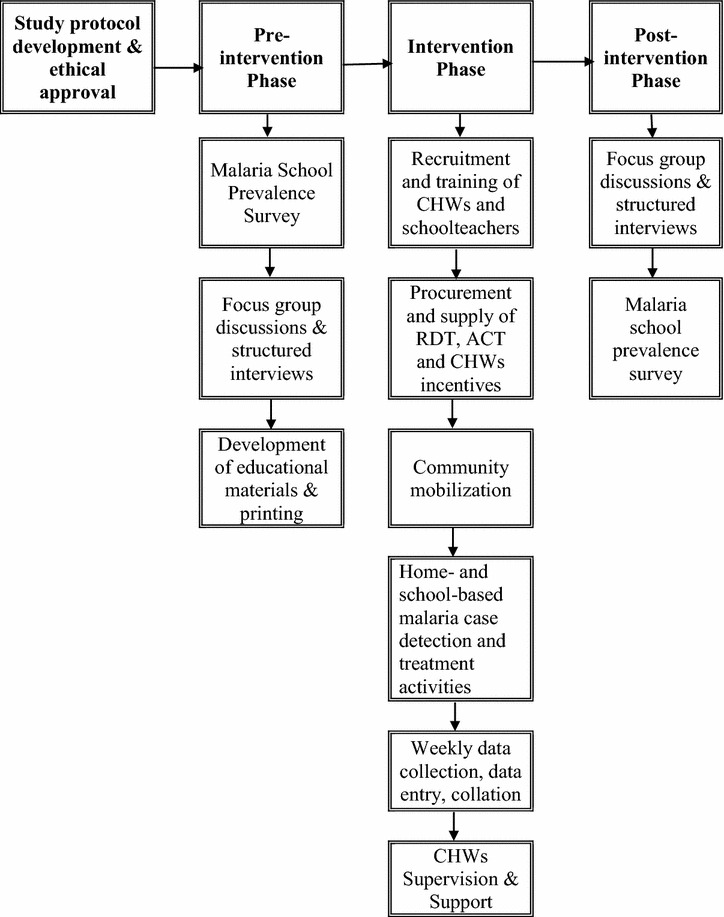
Study phases and main activity study flow

The investigation was a quasi-experimental design with one intervention (Mulumbu HA with CCMm) and one comparison area (Mpala HA without CCMm). The study was conducted during 13 consecutive months and was divided into three phases: pre-intervention, intervention, and post-intervention, respectively. Both quantitative and qualitative data collection was made in pre- and post-intervention phases to assess feasibility and impact of CCMm implementation. Figure 3 provides the general flow of study phases.

### Pre-intervention phase

#### Stakeholder’s involvement

Together with the study group, the Ministry of Health (Katanga Province), the National Malaria Control Programme (NMCP), the University of Lubumbashi, various international NGOs (Doctors without Borders, Family Health Association, C-Change FHI-360), United Nations affiliates (WHO, UNICEF) and the US President’s Malaria Initiative/United States Agency for International Development (PMI/USAID) in DRC were consulted for advice on study design and direct resource support before study implementation. After ethical approval for the study was obtained, the protocol was shared with stakeholders during a special Malaria Task Force meeting organized by the NMCP where expected challenges in study implementation were discussed. It was also agreed that the community would not pay any fees for provided services during the study and that all diagnostic testing and malaria treatment would be supplied free-of-charge.

#### Focus group discussions and structured interviews

The objective was to understand the general knowledge, attitude and practices regard childhood diseases with a focus on malaria. Focus group discussion (FGD) activities took place only in the CCMm intervention area.

Focus group discussions targeted mothers and/or close relatives who have or have had at least six children and/or were in charge of at least one child under 5 years-old at the time of participation. Prior to conduct FGDs, a deep review of what was published on the local communities (Sanga tribe) was performed and a field visit to refine questions of the FGDs guide was conducted. Findings indicated that in these communities, mothers who have or have had at least six children were able to report their own experience regarding children illness and treatment. They are also seen as a reference in the community.

A census of the 14 villages of the CCMm intervention area was conducted to identify the number of households per village and the number of members per household. A village was selected for inclusion in a FGD if there were at least 10 mothers who had or have had at least six children. In total nine villages full field this condition and were selected for FGD activities. In a village, mothers selection started with the ones who have or had have a very high number of children (means ‘a lot of experience’) up to those who have or had at least six children.

Focus groups were organized as structured discussions of 6–10 adults. They were conducted by experienced sociologists. To avoid any disruptions, FGDs were organized in a secured environment. A guideline was used with sub-questions and detailed notes were taken. In addition, consent for audio recording was obtained from mothers.

The following items were discussed: knowledge on infant illness and local believes, normal parental response to a child’s illness, preferred treatment-seeking behaviour, general access to treatment (e.g. formal health care facility, pharmacy, traditional, other), preferred medicine, general knowledge on malaria and its prevention, common or shared experience regarding malaria and history of previous malaria.

Focus group discussions were preceded with structured interviews of selected senior members of the village who had good knowledge of the village history. The objective of the interview was to understand the collective psychology (beliefs, attitudes, priorities, etc.) of the village elders, the interaction with the surrounding environment, and history and perceptions of malaria as a public health concern. In each of the nine selected villages four to five elders were selected for inclusion during interviews.

The following items were explored during interviews with village elders: origin of the village, first location of the village if any has happen up to the actual location and why, history of major outbreaks and children deaths and how people reacted. The interpretation of malaria as a public health concern was validated by the FGD that was organized the same day with ‘experienced’ mothers.

### Intervention phase

The intervention in Mulumbu HA consisted of five different sets of activities (1) selection and training of volunteers CHWs; (2) provide information, education and communication (IEC) in target communities; (3) procurement and supply of RDT and ACT; (4) training of CHWs on malaria case detection and treatment, and (5) routine study supervision and field support structure.

#### Selection, training and work activities of CHWs

The Mulumbu HA had no formal primary school before TFM constructed the initial facility in 2009. Consequently, many adults were illiterate and unable to write. Given these circumstances, the selection of CHWs was performed by the nurse-in-charge of the HA in conjunction with the various chiefs of the target villages under direct supervision of the FHZ staff. Selection was based on the following criteria: (1) having permanent residence in the village; (2) proven ability to read and write both French and Swahili (irrespective of having a formal professional diploma or degree, if any); (3) ability to work as a volunteer without receiving a set salary and prepared to spend the required time in testing and treating people regardless of time of day or night; (4) having a good and upstanding reputation in the target community (e.g., people with known drug or alcohol abuse issues were not selected); and (5) if married women, receiving a clear authorization from the husband to participate (as dictated by prevailing local customs). All CHWs that satisfied these conditions were introduced to the general community on a specific day for a final consensus approval by community members. During this event, the community was informed about the project objectives and that the appointed volunteers would be invited for a 3 day training session prior to study commencement. Three training modules were developed based on FGDs and structured interviews findings.

In total, in the Mulumbu HA, 25 CHWs (8 females and 17 males) and two nurses successfully completed the training session on malaria clinical signs and symptoms, diagnostic and case management based on current in-country NMCP guidelines. The training was carefully tailored to the group’s capabilities and focused on the identification of malaria infection using the RDT and subsequently treating all detected infections with the provided ACT. Additionally, special attention was also given for the identification of signs and symptoms of severe malaria infection using specific criteria guidelines on clinical presentation and providing pre-referral treatment (if possible) in the form of artesunate suppositories. This training also included identification criteria of other possible severe, non-malaria cases (e.g., measles, cholera) for referral to the nurse-in-charge of the Mulumbu clinic. Separate training was provided on use of a 35 × 45 cm malaria flip chart developed by the mine company MCP that also covered basic information on the biology and generic identification of malaria vector mosquitoes.

Each CHW was responsible for a set number (25–30) of households. CHWs periodically received sufficient quantities of ACT, RDTs, paracetamol tablets, thermometer to record axillary body temperature, notebooks, data tally sheets, and various stationery items to support their field work. They were also provided a bicycle to facilitate community coverage activities and movement to and from the health centre. As part compensation for their efforts during the study, each CHW was given the bicycle as personal property and a food basket at the end of the study.

#### Information, education and communication (IEC)

The day of the implementation of the CCMm, an extensive sensitization campaign using the malaria flip chart and jointly organized between the CCMm intervention HA Nurse-in-Charge and the FHZ staff was conducted with community residents and their leaders in each village. Scheduled events were announced 3–4 days before to ensure the largest participation possible. The community was shown and demonstrated the RDT kit, and provided answers to any questions or concerns raised by those in attendance. Subsequently, routine and regular IEC sessions were carried out by each CHW in their designated areas using one of the three modules and/or the malaria flip chart. Each CHW was requested to provide a weekly report that included the actual number of people contacted with the various forms of messaging.

#### RDT and ACT

Malaria RDT and ACT treatment supplies were provided by the PMI/USAID Integrated Health Programme. The RDT was a one-step, rapid, qualitative and differential test for the detection of HRP-II (histidine rich protein II) specific for *Plasmodium falciparum* and pLDH (*Plasmodium* lactate dehydrogenase) pan-specific sensitive to all human malaria parasite species (SD BIOLINE Malaria Ag P.f/Pan test, ref 05FK60, lot number 090158 from Standard Diagnostics Inc., Korea). For treatment of uncomplicated *falciparum* malaria, an artesunate-amodiaquine fixed dose combination (Batch number 1090, Winthrop^®^ Sanofi Aventis) as recommended by the DRC NMCP was used. For pre-referral treatment of complicated/severe cases, artesunate suppositories (Artesiane^®^ Suppogel, product number 11G27-B from Dafra Pharma GmbH, Tilburg, Netherlands) were available for administration for those unable to begin oral treatment. The standard NMCP malaria treatment criteria were applied for the clinical definition of complicated malaria and case management.

The bulk of study ACT medication was kept at the health facility level and all CHWs were supplied a field stock from the health facility on a periodic basis depending on use and need. A drugs procurement, distribution and recording system was developed for providing medicines in a timely manner at the community level and ensure that only high-quality (non-expired) drugs would be available throughout the study. The supply and distribution system was reviewed each day by the study Field Supervisor.

#### Home-based malaria case detection and treatment

Under direct supervision the CCMm was implemented from village to village on a gradual, systematic basis. After announcing the start date in a village, residents were requested to remain in the village that day. Initially, CHWs observe how the CCMm supervisory team performed the test followed by each CHW performing the test under direct supervision. All discrepancies in test technique and interpretation were corrected immediately. This exercise took between 2 and 4 days per village until the project team was confident that the CHW could perform all work independently, including documentation and reporting requirements before moving to the next village. Therefore, villages had varying periods of CCMm coverage compared to one another over the course of the trial. Once the CCMm was fully implemented in a village, local residents were requested to first seek CHWs for a malaria test when they had fever or other malaria-like symptoms.

The detection and treatment of malaria was based on an algorithm developed for CHWs, which took into consideration the result of the RDT as well as the other symptoms. The algorithm had four primary outcomes: (1) provision of ACT for treatment for uncomplicated malaria; (2) referral mechanisms for severe malaria cases including beginning pre-referral treatment, if necessary; (3) all cases of malaria infection (symptomatic or not) during pregnancy; and (4) referral mechanisms for apparent non-malaria cases. The following criteria were used for the basis of referring patients to higher level care: (1) patient with high fever (axillary temperature ≥37.5 °C) and RDT negative test results; (2) patient with high fever and RDT positive test with signs of complicated malaria (e.g., recent history of convulsions, repeated vomiting, evidence of impaired consciousness); (3) patient with RDT positive test with persistence of fever lasting >48 h after starting ACT treatment; (4) patient with RDT positive test that remained clinically ill after full (3 days) treatment with ACT; (5) patient that presented with worsening signs and symptoms at any time after beginning treatment; (6) pregnant women and infant of 2 months of age or less with RDT positive and (7) presentation of significant adverse side effects after taking medication (including nausea, vomiting, gastro-intestinal distress, allergic reactions). Symptoms suggestive of hepatitis, pre-icteric phase or jaundiced, and agranulocytosis (as suggested by a clinical condition including fever and/or tonsillitis and/or mouth ulcers) were to be carefully monitored. CHWs and the nurse-in-charge were instructed that when these symptoms developed or appeared exacerbated during the course of therapy, the patient should have treatment discontinued and be referred immediately to the referral hospital for laboratory tests on liver function profile and/or hematology.

Community health workers sent a weekly report to the nurse-in-charge. To ensure as best as possible completion of the full 3 day treatment course, CHWs supervised the first dose of treatment and requested the empty packaging with the name of the patient written on the package to be returned to them after 3 days. The health facility nurse collected all empty packs for periodic observation by the study Field Supervisor and all medical waste bins associated with the study for proper disposal and incineration.

#### Supervision and support

Study nurses based in the health facilities offered ongoing support to the CHWs. In each location, a field visit was conducted by the Field Supervisor each week, and when available, representatives from the FHZ coordination team participated. Every 3 months, all CHWs were assembled by the Principal Investigator and the FHZ representatives to share any difficulties and challenges encountered during the study.

### Post-intervention phase

At the end of the study, nine FGDs targeting mothers who have or have had at least six children, nine structured interviews targeting key people in the community (village elders) were conducted to compare with the results from the pre-intervention phase. In addition, three FGDs targeting 25 CHWs involved in the study implementation, and one structured interview targeting health care providers were conducted. The overall objective of these qualitative studies in post-intervention was to assess acceptability of the CCMm in the community and discuss challenges and programme improvements for the feasibility of expanding the CCMm in the FHZ. Appropriates questionnaires and guides were used.

#### Outcome measures

Two primary outcome measures were recorded as evidence of CCMm impact. (1) New malaria cases diagnosed and treated by community volunteers throughout the pilot study period, and (2) malaria school-based prevalence surveys (MSPS). The objective of the MSPS was to measure the malaria prevalence among a random sampling of schools and students aged from 6 to 12 years from primary schools located in the CCMm intervention and non-intervention HAs. This age group was considered highly representative and a geographically stable population for measuring malaria prevalence in the study sites. A stratified, randomized and proportional sampling method of students was used in line with previous school surveys conducted by the MCP by first randomly selecting schools and then randomly select students within each selected school. MSPSs used in the study were conducted in May 2012 and May 2013. The May 2013 school survey was the primary endpoint to measure the impact of the CCMm.

#### Data analysis

Data from CHWs was collected and collated from the field on a weekly basis and entered into a database (Microsoft Access 2010, Microsoft Corp. USA). Data from MSPS was collected on daily basis using Epi-info™, version 5. Data analysis was done using STATA version 14.1 software (Stata Corp LP, College Station, TX, USA). Inference tests were used to compare findings using proportional and Chi square analysis.

For qualitative studies in pre- and post-intervention phases, field audio recordings were transcribed and translated into French by the Faculty of Social Sciences, University of Lubumbashi in addition to detailed field notes written down under each sub-question of the focus group or structured interview guide. Pragmatical content analysis was performed to deduct the coherence, topics or themes with consensus, those with variance and those that definitely opposed. Structural homology theory [13] was used during findings interpretation to understand the knowledge, attitudes, practice and health-seeking behaviour, and common or shared experiences on malaria.

## Results

### Number and proportion of patients visiting CHWs

From November 2012 to August 2013, a total of 6619 contact cases with fever and other malaria-like symptoms were seen by 25 CHWs enrolled in the CCMm intervention HA. Population ages were ranged from 4 weeks to 83 years (median age of 10 years) and were grouped in four categories (Table 1): under 1 year, between one and 6 years, from 6 to 14 and over than 14 years. Children below 14 years of age represented 62 % of the total patients seen by CHWs. The sex ratio was 1.08 females for 1 male. Axillary temperature of all contact cases was ranged from 34.2 to 39.9 °C with a mean of 36.38 °C (SD ± 0.671).

**Table 1:** Age groups of contact cases during the CCM intervention phase

Age group (Years)	Gender (N = 6619)		Total (N = 6619)
	Female	Male	
<1, n (%)	95 (1.4)	90 (1.4)	185 (2.8)
1 ≤ 6, n (%)	1026 (15.5)	889 (13.4)	1915 (28.9)
6 ≤ 14, n (%)	1000 (15.1)	1003 (15.2)	2003 (30.3)
≥14, n (%)	1320 (19.9)	1196 (18.1)	2516 (38.0)
Total	3441 (52.0)	3178 (48.0)	6619 (100.0)

The average utilization by the target population over the 10 month project observation period was nearly 2 (1.97) contact-cases per CHW. This rate was estimated using the catchment population of 3343 as denominator and 6619 total visits to the CHWs. The average number of coverage households per CHW was 27, the equivalent of approximately 130 individuals each. During the 10 month study there was no requirement to replace CHWs.

### Number of malaria cases diagnosed and treated by CHWs

From the 6619 contact cases, 1433 individuals (21.6 %) were diagnosed with malaria infection using the RDT 74.7 % of this number were children below 14 years of age and infant under 1 year of age were 1.6 %. 737 cases (51.4 %) were females. However, there was no association between the sex and the positivity of the RDT (95 % CI 0.892–1.072, *p* = 0.978).

From this number, 1358 (94.7 %) did not present any signs of severe malaria, thus judged by the CHWs as simple, uncomplicated malaria cases. Nearly all (1354 uncomplicated malaria cases) received directly observed ACT on first day. Three cases did not get malaria treatment provided by CHWs. The first, a 15 year-old female presented with an acute adverse allergic reaction approximately 30 min after receiving the first dose of ACT and was immediately referred to the clinic for appropriate case management. With treatment, the case resolved quickly without further incident. Two other cases refused to complete the 3 day treatment regimen because of experiencing gastro-enteritis with vomiting and diarrhoea after taking the initial ACT dose. They were also referred to the clinic for more advanced case management. Both patients fully recovered without further incident.

### Patients referred by CHWs to the clinic

278 patients (4.2 %) were referred to the CCMm clinic by CHWs based on established criteria. From this number, 89 (32.0 %) had RDT positive and 189 (67.9 %) had RDT negative tests results.

61/89 RDT positive patients presented clinical signs of severe malaria including 44 children who received artesunate suppository as a pre-referral treatment and two pregnant women. From the remaining 28 patients with RDT positive, 22 were asymptomatic pregnant women. 132/189 RDT negative patients were referred because of fever (axillary temperature ≥37.5 °C). There was as significant association between fever and RDT positive (95 % CI 0.285–0.345, *p* = 0.314) and the risk was three times (prevalence ratio 3.188 ranged between 2.898 and 3.507).

### Supervision

In total, 274 field supervisory visits were conducted during 41 weeks of observation to assist the CHWs, to ensure that study protocols were being observed, and take stock of RDTs and ACT medication, both at the source clinic and community distribution points. These visits also allowed the controlled collection and proper disposal/treatment of biomedical waste (e.g., used RDTs, blood lancets, etc.) generated during this study.

### Outcome measures

#### Malaria prevalence

Data was provided from two malaria school prevalence surveys (MSPS) conducted in the intervention and comparison areas in May 2012 before the CCMm intervention began and as a primary outcome measure in May 2013 at the trial’s conclusion. The MSPS were a routine monitoring component of the integrated MCP inside the concession coverage area and were independent of the CCMm trial.

**Fig. 4:**
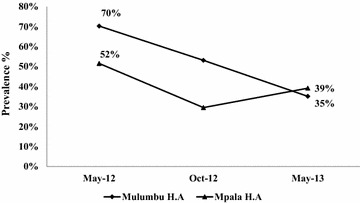
May 2013 school-based malaria infection prevalence at post-intervention in the CCMm intervention area (Mulumbu HA) was not statistically different from the non-CCMm area (Mpala HA)

In pre-intervention, the malaria prevalence rate in sampled CCMm school children was 70.3 % [range 57.6– 81.1] and 51.6 % [range 42.5–60.6] in the comparison coverage area. The malaria prevalence at the beginning of the study was significantly higher in the CCMm HA compared to the comparison HA (χ^2^ 6.105, *p* = 0.0135). At post-intervention, the May 2013 survey found malaria prevalence in the intervention zone had declined to 35.1 % [24.4–47.1], while declining to 39.2 % [30.8–48.2] in the comparison area. There was no statistical difference in malaria prevalence (χ^2^ 0.336, *p* = 0.562) between the CCM intervention and comparison schools after the 10 months of CCMm intervention. However, within the CCM intervention HA, malaria prevalence decreased significantly in post intervention compared to the pre-intervention (χ^2^ 17.00, *p* = 0.000039) and the risk was divided by two (prevalence ratio = 2.00 ranged between 1.4129 and 2.8345) (Fig. 4).

#### Malaria incidence

**Fig. 5:**
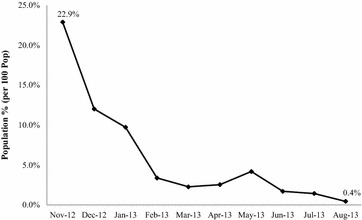
Malaria infection rate from cases detected by CHWs in Mulumbu Health Area at beginning (Nov 2012) to end (Aug 2013) of CCMm intervention period

All new malaria cases diagnosed and treated by CHWs were recorded throughout the pilot study period. The implementation began in seven villages in November 2012 and gradually included all 14 villages. Monthly malaria incidence rates per 100 population were calculated. The monthly malaria incidence went from a maximum of 22.9 % in November 2012 to 0.4 % in August 2013 (Fig. 5).

#### Focus group discussions in pre- and post-intervention periods

Nine FGDs targeting mothers that met inclusion criteria were conducted during post-intervention in nine selected villages out of 14 to compare FGDs results performed in the same villages before intervention. In total 87 mothers were included and four themes were discussed to arrive at consensus agreement among participants.

*Theme 1 Childhood diseases: knowledge and practice*. In the pre- and post-intervention, the majority of mothers believed that the top three life threatening diseases in children under 5 years-of-age were malaria, measles and diarrhoea. Malaria was regarded the most dangerous because it was perceived as the greatest risk of causing severe fever, anaemia, coma and premature death. In the pre-intervention phase, FGDs responded that malaria was either transmitted by a mosquito bite or by drinking dirty water or the result of having an enlarged spleen (actually the consequence of malaria infection) called “*kipima*” in the local language. This information and beliefs were commonly transmitted by parents to children over generations. In the post-intervention phase, practically all mothers agreed that a mosquito bite was responsible for transmitting malaria although a few retain the notion that *kipima* was also the cause. However, no one responded that contaminated water could result in malaria in the follow-up. All responded that they received the information on malaria transmission through periodic instruction provided by local CHWs using the malaria flip chart.

*Theme 2 Mothers attitudes regarding malaria in children.* In the pre-intervention phase, when a child was sick, the majority of mothers responded that they started by giving the child traditional treatments based on local herbs, roots and other plant parts. A list of 15 herbs, roots and plants used locally to treat malaria (or what was perceived to be effective) was provided. Sometimes a traditional treatment was combined with ‘modern’ medicine, primarily paracetamol tablets to reduce fever. When the health condition of child became worse, they would seek alternative treatment at the nearest health facility. In the post-intervention phase, the majority of mothers responded that when a child is sick they would seek first the CHW in their village for a blood examination and treatment for malaria if infected.

In pre- and post-intervention, mothers decide first on the treatment to be given to the ailing child to include seeking modern treatment and providing the prescribed drugs. Fathers were often not involved and typically far removed from the child rearing duties. In pre-intervention, the majority of mothers responded that they would consider stopping the administration of prescribed drugs if the child felt well before the end of the full course of treatment, while in the post-intervention follow-up, the majority of them emphasized the importance of completion of the full course of treatment regardless of child’s apparent recovery from infection.

*Theme 3 Practice toward malaria prevention and treatment.* In the pre-intervention, when asked “What can you do to prevent malaria?” the majority of mothers believed that nothing could be done—malaria was more or less inevitable and an element of fate. On the other hand, by the end of the study, the vast majority mentioned three methods that could be useful to prevent malaria: sleeping under a bed net, allowing their house to be sprayed with an insecticide (IRS), and preventing stagnant water to collect near the house. In one village, mothers stated that the “*efficacy of a bed net depends on the way that it is set*-*up and how it is used. If the net has holes or not properly used (e.g., ends tucked under the matt), mosquitoes can be in contact with children and transmit malaria*”. The majority of mothers in post-intervention preferred to treat their children with modern drugs (namely ACT) as they perceived its efficacy as far better compared to traditional treatments.

*Theme 4 Experience regarding malaria and history of previous malaria.* In the pre-intervention, all mothers had at least one child with history of malaria (confirmed or suspected) the previous 6 months. All reported having knowledge that children had died in their villages in the last 6 months because of malaria. In the post-intervention, all mothers reported there was no child deaths in their villages attributed to malaria during the duration of the study.

#### Structured interviews targeting key people in the community

Key community leaders participated in nine structured interviews, one in each selected village with at least four key community leaders gathered together in pre- and post-intervention. The purpose was to understand their perception on the CCMm, the work performed by CHWs, and the main challenges faced during the implementation of the CCMm. In the post-intervention, all reported that CHWs did a very good job in providing a proper malaria diagnosis using RDTs and subsequently treating infections. Others noted that CHWs conducted useful education sessions and quickly referred cases to the local health care facility that they could not effectively manage. The biggest benefit perceived by community leaders was a significant reduction of malaria cases in children. The most significant challenge or concern was that CHWs lacked financial compensation for the additional work in covering the designated CCMm households in the village.

#### Focus group discussions targeting CHWs

Three FGDs, two of eight participants and the third of nine participants were organized. All 25 CHW participants reported that they were satisfied with their additional roles and responsibilities in their respective villages. They received in return the respect and acknowledgement from the communities they served. CHWs confidently reported that people in the community would consult them first because of increased knowledge on malaria and the convenience of providing immediate free-of-charge malaria diagnosis and treatment. Many suggested that in future their scope of work would benefit more by adding other illness conditions such as management of diarrhoea and other febrile no malaria illness. In addition to the bicycle and food basket provided to the CHWs all requested some form of direct financial compensation for time not spent doing normal activities.

#### Structured interviews of health care providers

Three nurses from the two clinics in the CCMm intervention HA were interviewed: two were previously trained with one acting as the nurse-in-charge of the HA and the third was recruited during the pilot study implementation. They reported each receiving an average of nine patients per month referred to by the CHWs. All referrals were reviewed, the majority of which were deemed appropriate based on set referral criteria. The major reasons for referral were: malaria in children under 5 years of age, cases meeting ‘severe’ malaria criteria, other conditions like malnutrition, upper respiratory tract infections or gastroenteritis, patients RDT negative with fever, and patients RDT positive with persistent fever after 3 days of treatment. They also reported that some patients referred by CHWs did not come to the clinic because treatment was not provided free-of-charge at the clinics unlike in the CCMm setting. All agreed that there was a significant reduction of malaria cases during the study period, but added it caused a notable reduction of clinic income normally generated from user fees. Malaria was recognized as the primary reason for medical consultation and thus contributing significantly in generated monies for the maintenance of the rural clinics.

## Discussion

This combined qualitative and quantitative pilot study explored the feasibility and impact of implementing a community-based malaria diagnostic and treatment by trained and supervised CHWs as argumentation to an existing integrated malaria control programme in DRC. At pre-intervention, baseline malaria prevalence was significantly higher in the intervention zone compared to the comparison area. After the intervention, comparisons between the two areas indicated no statistical difference in malaria prevalence based on respective area school surveys. The authors acknowledge that randomized school surveys may provide only anecdotal evidence of direct impact by CCMm but still regard the school-based method of sampling as evidence-based and reflective of risk and transmission in each respective area. Although CCMm and non-CCMm areas were not directly comparable (differing population size and number of villages covered), the comparisons are generally valid as both locations were receiving the same malaria control interventions (IRS and LLINs) and both areas, in general, would have been impacted by the prevailing dry season effects which typically show a natural reduction in malaria risk and disease leading up to the next wet season. The only meaningful difference between the two areas was the introduction of the CCMm in the Mulumbu HA. The present findings suggest that the reduction in malaria in Mulumbu was at least partly due to the CCMm and that over time the accumulated impact of the activity would have been even greater. The authors admit that a limitation to drawing definitive conclusions on CCMm impact was the relatively short time frame of observation (10 months) and small population size covered in this pilot study.

The approximate community utilization rate throughout the 10 months found, on average, each person in the 14 CCMm villages sought assistance twice from a CHW regard a malaria RDT and treatment, if required. This indicates acknowledgement, adherence and acceptability of the CCMm strategy by the targeted population. This acceptability of the CCMm was also corroborated from the FGDs findings of mothers regard malaria treatment-seeking behaviour and from the structured interviews of community leaders. Several possibilities could explain these findings. First, the implementation of the CCMm in all villages was based on the results of the pre-intervention qualitative studies where the background knowledge and practices of the targeted communities were assessed. From this, the diseases of greatest concern and those responsible (or perceived) for causing the greatest number of deaths (i.e., malaria) were identified, followed by messages to sensitize the community to the benefits of the CCMm strategy. Secondly, detailed training and testing was provided to all CHWs, followed by a systematic village-by-village CCMm programme implementation. CHWs were allowed to independently perform the RDT and provide ACT only when the study team was confident with their performance. Thirdly, regular supervision visits by the study team to observe and correct any discrepancies in trial protocols and procedures served as an additional opportunity to interact more closely with the communities. It was also acknowledged that because the testing and treatment were both convenient and free-of-charge, community members were far more likely to utilize this service compared to the cost (transport and clinical care) and additional time associated attending the local clinic.

Community acceptability of a CCMm has been assessed in different African countries. Studies in Uganda [14] and in Cameroon [15] took into account the opinion of community members before introducing an RDT-based CCMm intervention. In Uganda, presumptive CCMm had already been implemented with a positive community attitude towards the CHWs due to their voluntary service, ease of accessibility and effectiveness of service [14]. The community also welcomed the change to using RDTs for malaria diagnosis as an easier and quicker process for detecting infection and treatment. In an urban area of Cameroon where alternative healthcare providers are available, CHWs were not always considered to be the appropriate persons to carry out RDTs. However, in both studies, participants stressed that proper training of CHWs on RDT use was essential for the programme to be accepted and utilized by the community. After introduction of a RDT-based CCMm in Uganda, community members were asked their opinion of the programme [16]. The majority of respondents (79.4 %) believed the CHWs’ service was better after introduction of RDTs and 88.7 % thought CHWs should continue to use RDTs as the basis for malaria treatment. In Senegal [17], investigators also found a high acceptance of a CCMm where community members praised the increased access to malaria care.

In the DRC study, each CHW was responsible for an average of 27 households, the equivalent of around 130 residents. In this rural setting, this coverage responsibility appeared reasonable and manageable as no CHW complained about the workload during the post-intervention FGDs. In addition, CHWs were selected as volunteers, agreed upon by consensus by the local community, and had initially accepted to provide services without direct compensation. This too could play a big role regarding community acceptability and programme sustainability over time. However, later into the programme, all CHWs inquired about the possibility of some form of financial compensation as did some of the community leaders on their behalf. Although an understandable request, the expectations were likely linked to the fact that the CCMm study was supported by the major employer (mining operation) in the area. Any additional and substantial recurring costs associated with a community-based ‘volunteer’ CHW network could greatly impact programme expansion and sustainability.

Findings from qualitative post-intervention interviews and FGDs involving CHWs, nurses and community leaders supported the acceptance of the CCMm strategy. Overwhelmingly, the communities welcomed the CCMm because of its positive impact in more quickly diagnosing and treating malaria and a further perception in reducing the number of malaria cases in the community during the observation period. Respondent mothers reported that for the first time there were no children deaths attributed to malaria in any of the 14 target villages. Albeit, qualitative data was mostly anecdotal but viewed as a very promising response, the relatively short period of study observation (less than 1 year) was a limiting factor in understanding the full, longer-term epidemiological impact of CCMm on malaria.

Community education using a specially designed, site-specific, malaria flip chart and other educational tools clearly improved the level of community knowledge and practice on control of malaria. Responses at the end of the study indicated that the majority of mothers believed that a mosquito bite was the primarily cause for transmitting malaria (as opposed to dirty water) and that it was important to take the full course of malaria treatment even when a patient feels better before all drugs are consumed. Mothers also indicated that they would first seek the local CHW for providing rapid diagnosis of suspected malaria and treatment, rather than using traditional therapies involving local herbs, roots and various other plant products, a belief and practice that was more common before the intervention began. However, behaviour change and understanding takes time, patience and repeated interaction [18]. The findings indicated that there were still individuals that believed malaria can be transmitted from having an enlarged spleen (rather than the cause of the condition). This indicates that repeated community education is a long-term endeavour in order to effect permanent behavioural change and dispel incorrect perceptions and is an important component to successful implementation of a CCMm strategy.

The perceived efficacy of ACT by the community as the best means to treat malaria is a critical step to ensure CCMm can have a positive impact in reducing malaria morbidity and mortality. A gradual lowering of the number of malaria infected human reservoirs in a population will theoretically translate in a reduction in malaria transmission risk over time. The premise that reduced probability of infected/infectious mosquitoes because of fewer infectious human malaria reservoirs would result in a reduction in infection risk (disease incidence). However, this may not necessarily translate in a measured reduction in malaria prevalence over a short term.

Malaria rapid diagnostic tests have the potential to greatly reduce the overtreatment of malaria, upwards by 95 % [19]. The development of accurate RDTs that can be used in basic healthcare services in remote settings led WHO to recommend a switch to universal parasitological testing before treatment in 2010 [20]. During the CCMm implementation phase, 67.9 % of patients referred by CHWs to the clinic (189/278) had RDT negative tests results and among them 132 (69.8 %) were referred because of fever (axillary temperature ≥37.5 °C). The authors acknowledge that fever may be caused by a number of reasons. However, in the settings of low prevalence, the reliance on RDTs for diagnosis, although superior to a purely clinical diagnosis alone, will miss some (e.g., low density) infections or may otherwise indicate an infection that does not exist (false positive result) [19, 21]. Together with asymptomatic infections that do not seek health care or those self-treating for malaria infection using a substandard treatment will remain problematic ‘silent’ sources of parasites to further transmission in an area. To overcome this, one possible alternative to the standard RDT would be to introduce field-ready, real-time PCR-based technologies such as a photo-induced electron transfer-PCR [22, 23] or a loop-mediated isothermal amplification [24, 25]. Such devices have increased sensitivity and specificity to detect much lower density *plasmodia* infections, thus capture a greater number of infected persons for treatment. Another possible option would be to introduce point-of-care rapid detection devices for glucose-6-phosphate dehydrogenase (G6PD) enzyme deficiency in malaria-infected patients [26]. Those found with normal G6PD enzyme activity could be given a single, low dose amount of primaquine that would effectively neutralize infectivity of *Plasmodium* gametocytes in vector mosquitoes [27]. Theoretically, this would have a greater impact in lower malaria-endemic settings, but nonetheless, as the percent of malaria reservoirs decline, so will the number of infective vectors that result in secondary cases.

Based on interviews and responses, financial concerns of CHWs may be a major challenge for a scaling up of the CCMm in the FHZ and elsewhere in DRC. Based on post-intervention FGDs, community incentives provided throughout the study period did not suffice CHW’s expectations as sufficient compensation for extra duties preformed. All expressed the desire of receiving some direct financial support for their efforts. Additional approaches used in other countries such as Uganda, Mozambique, Tanzania and Malawi [28–32] need to be explored and adapted site-specific before any decision for scaling up CCMm in the FHZ can be implemented. Techniques encouraging positive behavioural change and community empowerment might be one approach to gain local support for volunteers exclusive of direct compensation from external sources [33]. Further areas for research include the cost-benefit analysis of a CCMm per patient diagnosed and treated versus the cost incurred at a local primary health care facility. In other words, the cost of scaling up a CCMm may be outweighed by simply increasing convenience and access to established clinics even if there are costs associated with diagnosis and treatment at private and government health care facilities.

## Conclusion

As part of integrated malaria control programme, the first feasibility and implementation trial of CCMm using trained volunteer CHWs with RDT-based diagnostics and ACT in southern DRC was shown to be an acceptable approach for detecting and treating infections promptly and effectively. Although the study provides only anecdotal or surrogate findings on impact, it remains a promising strategy to improve basic medical access to rural, underserved populations regard prompt and effective management of malaria. Encouragingly, all villages involved in the CCMm intervention expressed high acceptability of the approach and indicated that the programme resulted in a reduction of both malaria morbidity and mortality during the implementation phase. In addition to other control methods in the area (IRS and LLINs), if CCMm were to be scaled-up to serve a greater number of more remote village communities suffering from perennially high, near intractable malaria transmission, this additional method would potentially contribute to a significant decrease in malaria morbidity and mortality over time. Further refinement of CCMm implementation, cost implications and sustainability of scaling up this community-based intervention in DRC is encouraged.

## Abbreviations

ACT: artemisinin-based combination therapy
CCMm: community case management of malaria
CHW: community health worker
DRC: Democratic Republic of the Congo
FGDs: focus group discussions
FHZ: Fungurume health zone
HA: health area
IEC: information education and communication
IRS: indoor residual spraying
LLINs: long-lasting insecticidal nets
MpHA: Mpala health area
MuHA: Mulumbu health area
NMCP: National Malaria Control Programme
PMI: United States President’s Malaria Initiative
RDT: rapid diagnostic test
TFM: Tenke Fungurume Mining
UNICEF: United Nations Children’s Fund
USAID: United States Agency for International Development
WHO: World Health Organization
